# Exposure to parasitic infections determines features and phenotypes of active convulsive epilepsy in Africa

**DOI:** 10.12688/wellcomeopenres.17049.1

**Published:** 2021-08-05

**Authors:** Nelson K. Langat, Symon M. Kariuki, Gathoni Kamuyu, Angelina Kakooza-Mwesige, Seth Owusu-Agyei, Kenneth Ae-Ngibise, Anne Wang'ombe, Anthony K. Ngugi, Honorati Masaja, Ryan G. Wagner, Charles R.J.C. Newton, on behalf of the Study of Epidemiology of Epilepsy in Demographic Sites (SEEDS) group

**Affiliations:** 1 Clinical-Neurosciences, Kenya Medical Research Institute (KEMRI)/Wellcome Trust Research Programme, Kilifi, Kenya; 2 Institute of Tropical and Infectious Diseases, University of Nairobi, Nairobi, Kenya; 3 Department of Public health, Pwani University, Kilifi, Kenya; 4 Department of Psychiatry, University of Oxford, Oxford, UK; 5 Department of Paediatrics and Child Health, MakerereUniversity College of Health Sciences, Kampala, Uganda; 6 Kintampo Health Research Centre, Kintampo, Ghana; 7 Institute of Health Research, University of Health and Allied Sciences, Ho, Ghana; 8 Department of Population Health, Medical College (East Africa), Aga Khan University, Nairobi, Kenya; 9 Ifakara Health Institute, Ifakara, Tanzania; 10 MRC/Wits Rural Public Health & Health Transitions Research Unit (Agincourt), School of Public Health, Faculty of Health Sciences, University of the Witwatersrand, Johannesburg, South Africa

**Keywords:** Epilepsy, parasitic infections, status epilepticus, neurologic deficits, intellectual disabilities, seizures, Africa

## Abstract

**Background**: Epilepsy affects 70 million people worldwide, 80% of whom are in low-and-middle income countries (LMICs). Infections of the central nervous system (CNS) contribute considerably to the burden of epilepsy in LMICs, but the nature and presentation of epilepsy following these infections is not fully understood. We examined if epilepsy features and outcomes are associated with the exposure to parasitic infections.

**Methods**: This was a case-comparison study nested in a cross-sectional survey of people with active convulsive epilepsy, with cases as those exposed to parasitic infections, and comparison as those unexposed. Associations of exposure to parasites with clinical and electroencephalographic features of epilepsy were done using a modified mixed effects Poisson regression model across five sites in Africa. Multiplicative and additive scale (RERI) interactions were explored to determine the effect of co-infections on epilepsy features. Population attributable fractions (PAF) were calculated to determine the proportion of severe clinical and electroencephalographic features of epilepsy attributable to CNS infections.

**Results:** A total of 997 participants with active convulsive epilepsy from the five African sites were analyzed, 51% of whom were males. Exposure to parasitic infections was associated with more frequent seizures in adult epilepsy (relative risk (RR)=2.58, 95% confidence interval (95%CI):1.71-3.89). In children, exposure to any parasite was associated with convulsive status epilepticus (RR=4.68, (95%CI: 3.79-5.78), intellectual disabilities (RR=2.13, 95%CI: 1.35-3.34) and neurological deficits (RR=1.92, 95%CI: 1.42-2.61). *Toxoplasma gondii* and *Onchocerca volvulus* interacted synergistically to increase the risk of convulsive status epilepticus (RERI=0.91, 95%CI=0.48-1.35) in the data pooled across the sites. Exposure to parasitic infections contributed up to a third of severe features of epilepsy as shown by PAF.

**Conclusions:** Parasitic infections may determine features and phenotypes of epilepsy through synergistic or antagonistic interactions, which can be different in children and adults. Interventions to control or manage infections may reduce complications and improve prognosis in epilepsy.

## Introduction

Epilepsy is a common serious neurological disorder, with an estimated 70 million people being affected, 90% of whom live in low-and-middle-income countries (LMICs) ( Ngugi *et al*., 2010). Global incidence of epilepsy is up to 278 per 100,000 people, while global prevalence is up to 11 per 1,000 people ( Fiest *et al*., 2017), with both incidence and prevalence being higher in LMICs than high-income countries (HICs) ( Newton & Garcia, 2012). The higher burden of epilepsy in LMICs can be attributed to the higher incidence of infections such as malaria and human immunodeficiency virus (HIV), birth-related injuries and low accessibility to health care ( Newton & Garcia, 2012).

A study carried out in selected African populations indicated that exposure to multiple parasites was associated with the risk of epilepsy ( Kamuyu *et al*., 2014). However, this study did not determine the contribution of infections to features and phenotypes of epilepsy and examine response to anti-seizure medications (ASM) ( Kariuki *et al*., 2014). It is important to describe the features and phenotypes of epilepsy ascribable to exposure to infections since most infections are preventable through simple public health interventions. Infections such as malaria and HIV can increase the risk for status epilepticus ( Kariuki *et al*., 2015a) and frequent seizures, both of which worsen prognosis of epilepsy. Early onset epilepsy, which is common in Africa, may be caused by infections, which affects children because of undeveloped immunity and brain. Infections may also damage the brain resulting in comorbidities of severe epilepsy such as intellectual disability and neurological deficits, which can affect adherence to ASM ( Ibinda *et al*., 2017; Mbuba *et al*., 2012). Additionally, exposure to infections may complicate biomedical treatment of epilepsy. It important to determine the burden of severe epilepsy that could be prevented by control of infections. Findings from such studies may be useful in informing policy makers on the impact that control of these infections may have on the burden of epilepsy, particularly towards efforts to reduce the clinical and psychosocial burden of epilepsy.

We performed an analysis of people with epilepsy with well-defined clinical features to examine if exposure to common central nervous system (CNS) infections may play a role in the early onset of epilepsy, convulsive status epilepticus, frequent seizures, abnormal electroencephalograph (EEG) findings, intellectual disabilities, neurological deficits as well as other medical comorbidities of epilepsy e.g. malnutrition. We computed the proportion of severe epilepsy that is attributable to these infections, which allows us to estimate the clinical and psychosocial burden of epilepsy that would be eliminated following the control of these infections.

## Methods

### Ethical considerations

The study was approved by the ethics committees or ethics review boards in each of the participating countries (Clearance number: M080455 for Agincourt, South Africa; IHI/IRB/No A 70-2009 for Ifakara, Tanzania; Reference number: HS 663 for Iganga, Uganda; SSC number 787 for Kilifi, Kenya; FWA number 00011103 for Kintampo, Ghana;). All participants or guardians gave written informed consent before responding to study-related questions.

### Study site and population

The study was carried out in sites in five African countries with Health Demographic Surveillance Systems (HDSS), which are part of the International Network for the Demographic Evaluation of Populations and Their Health (INDEPTH): Agincourt, South Africa; Kintampo, Ghana; Kilifi, Kenya; Ifakara, Tanzania; and Iganga-Mayuge, Uganda. These sites collect health and vital demographic data from residents on a regular cyclical basis. The study population for each HDSS was described in detail previously ( Ngugi *et al*., 2013).

### Study design

People with epilepsy were identified from a 3-stage community cross-sectional survey carried out between 2008 and 2011 ( Ngugi *et al*., 2013). Screening for history of convulsions was conducted in stage I, followed by administration of a detailed questionnaire to describe seizures in stage II, and a neurological assessment by a clinician to diagnose epilepsy in stage III. Formal validation of this three-stage methodology is published elsewhere ( Ngugi *et al*., 2012), and findings supported need for continued efforts to develop and improve case-ascertainment methods in population-based epidemiological studies of epilepsy in LMIC. Community members diagnosed with epilepsy were tested for antibodies to HIV and infectious parasites ( *Toxacara canis, Onchocerca volvulus, Plasmodium falciparum, Toxoplasma gondii,* and *Taenia solium)* and formed the sample for this case-comparison study. The exposure was any of the listed infections and the outcomes were the presenting features of epilepsy. All study materials can be found as extended data ( Langat *et al*., 2021).

### Sampling

The population consisted of 1,711 patients diagnosed with epilepsy in the five sites. Respondents were tested for *Toxacara canis, Onchocerca volvulus, Plasmodium falciparum, Toxoplasma gondii,* HIV and *Taenia solium* (exposed). HIV was tested because it is often cormobid with these parasitic infections. Sample size calculation showed this study needed 386 participants to determine the association between features of epilepsy and infections at 80% power and 95% level of confidence (supplementary appendix 1). However, we utilized all data available (997 patients) in this study.

### Procedure

***Clinical assessments and definitions.*** Most clinical and EEG features, treatment and detrimental consequences were obtained through clinical examination and EEG recordings performed by trained clinicians, and questionnaires. The clinical and neurophysiological features of epilepsy have been reported previously ( Kariuki *et al*., 2014; Kariuki *et al*., 2015b). Epilepsy was defined as ≥2 unprovoked seizures occurring at least 24 hours apart and was classified as active if at least one seizure occurred within the previous 12 months ( Thurman *et al*., 2011).

Briefly, convulsive status epilepticus was defined as seizures lasting >30 minutes or intermittent seizures lasting for 30 minutes without recovery of consciousness. Seizure frequency was categorized into daily (at least one each day), weekly (at least one a week), monthly (at least one a month), and yearly (at least one a year). An EEG was categorized as abnormal if there was evidence of an abnormal background, focal changes, interictal epileptiform activity or an abnormal response to either of the activation procedures (hyperventilation or photic stimulation). Seizures were classified as focal, generalized, or other. Intellectual disability was assessed by a clinician observing young children who had problems performing the standardized test of a locally adapted developmental inventory, or by assessing an adult’s awareness of person, place and time. Malnutrition was defined as a weight-for-age *z* score value of −2 or lower or a mid-upper arm circumference less than 11.5 cm. Neurological deficits were defined as an inability to do things and walk or sit upright if of an appropriate age. Severe epilepsy was considered in a person with epilepsy plus focal seizures, frequent seizures, abnormal EEG, neurological deficits or intellectual disability.

***Serological assay analysis.*** Data on assays for parasitic and HIV infections were obtained from the work by Kamuyu and colleagues ( Kamuyu *et al*., 2014). They determined infection status through analysis of blood samples to detect antibodies to the infectious parasites and HIV. The kits and techniques used, criteria for cut-off and sensitivity and specificity have been described ( Kamuyu *et al*., 2014). These assays were performed on 986 people with epilepsy.

### Data management and statistical analysis

The data was exported from MS Excel to STATA version 15 for sampling and analysis. Data cleaning was done to assess completeness of data and exclude duplicates. For continuous data (e.g. age), histograms were plotted to show distribution. Mean (standard deviation (SD))/median (Inter-quartile range (IQR)) were reported depending on the nature of the distribution of data (i.e. whether or not the data is normally distributed). Histogram plots and Shapiro-Wilk’s test of normality were performed to check for normality of data, followed by transformation where appropriate. Categorical data such as sex, education, HIV status were illustrated using bar charts, with associated frequencies and proportions presented in tables.

Student’s t-test was used to compare means of titers of infection in those with severe features of epilepsy and those without, if the raw or transformed data was normally distributed. For non-parametric continuous data, Mann-Whitney U test was used. The association between parasitic infections (predictors) and features of epilepsy (outcomes) was determined using a mixed effects modified Poisson regression model with robust standard errors. Since our outcomes are binary, using robust standard errors rectified overdispesion and produced reliable estimates. Modified Poisson regression model was chosen over log-binomial model for two reasons: (i) it is not largely affected by convergence problems, and (ii) it allows for addition of random effect(s) variable while log-Binomial model does not. The model uses a log function to link a binary outcome (feature of epilepsy) to a set of predictors (infections and confounders), while accounting for clustering effect of site. To obtain crude relative risks, simple modified Poisson regression model with site as a random effect was used. Adjusted relative risks were obtained using multiple modified Poisson regression models with site as a random effect. We chose relative risk over odds ratio (OR) since the prevalence of some features was >10%, to avoid exaggerating risks with OR. In this model, the outcomes were the various clinical, or neurophysiological features of epilepsy (focal seizures, status epilepticus, frequent seizures [daily/weekly], neurological deficits, abnormal EEG, cognitive difficulties, malnutrition) and predictors were the parasitic infections most commonly associated with epilepsy ( *Toxacara canis, Onchocerca volvulus, Plasmodium falciparum, Toxoplasma gondii,* T *aenia solium, and HIV (included because increases risk for these parasites)*) combined or considered individually together. The model was accounted for age, sex, other socioeconomic confounders and ASM use. Biological (additive) interaction was assessed to determine public health implications of co-infections on features of epilepsy. Biologic interaction is more informative than the multiplicative interaction in the sense that the former gives the additive effect (synergism/antagonism) of exposures on the feature of epilepsy, by determining the relative excess risk due to interaction (RERI). RERI enabled us to know whether the combined effect of the exposures exceeds (synergism) or is less than (antagonism) the effect of each considered individually. RERI was determined as follows:

Additive interaction (RERI) = ( *p*_11_– *p*_00_)–[( *p*_10_– *p*_00_)+( *p*_01_– *p*_00_)]        (1)

Where;

*p*_00_- Prevalence of the epileptic feature when both infections are absent; *p*_11_- Prevalence of epileptic feature when both infections are present; *p*_10_ and *p*_01_- Prevalence of epilepsy feature when one infection is present.

## Results

### General description

A total of 997 participants with active convulsive epilepsy from the five African sites were included in the analysis. The overall proportion of males was 51% across all the samples, ranging from 48% in Ifakara to 56% in Agincourt. The overall median (IQR) age was 20.0 years (13.0 – 32.5), being lowest in Iganga (12.0, IQR: 5.0–21.0) and highest in Agincourt (29.0, IQR:18.0-45.0). There was a significant difference in age (P=0.0001) across the sites (supplementary figure 1). The distribution of these and other sociodemographic characteristics including education and employment status are shown in Table 1 and most characteristics varied by site.

**Table 1.** Socio-demographic characteristics and comorbidities of study population by site.

	Agincourt	Ifakara	Iganga	Kilifi	Kintampo	Total
	n = 176	n = 287	n = 84	n = 276	n = 174	n = 997
**Socio-demographics**						
Median age in years (IQR)	29.0 (18.0–45.0)	18.3 (12.0–30.9)	12.0 (5.0–21.0)	18.0 (11.0–27.5)	21.0 (15.0–29.0)	20.0 (13.0–32.5)
Median age (years) at onset of seizures (IQR)	13.7 (2.6–31.1)	8.9 (2.0–16.5)	2.1 (0.5–7.9)	3.3 (1.0–13.4)	9.8 (3.0–16.8)	6.7 (1.6–16.8)
Sex; n (%)						
Female	78(44.3)	150(52.3)	40(47.6)	143(51.8)	78(44.8)	489(49.0)
Male	98(55.7)	137(47.7)	44(52.4)	133(48.2)	96(55.2)	508(51.0)
Education (age≥5); n (%)						
Schooled	121(71.6)	160(58.6)	43(63.2)	153(60.5)	107(62.6)	584(62.6)
Unschooled	48(28.4)	113(41.4)	25(36.8)	100(39.5)	64(37.4)	350(37.5)
Employment (age≥18); n (%)						
Employed	7(5.3)	106(72.6)	4(16.7)	42(30.9)	54(47.8)	213(38.6)
Unemployed	126(94.7)	40(27.4)	20(83.3)	94(69.1)	59(52.2)	339(61.4)
**Co-morbidities**						
Neurologic deficits; n (%) No	139(79.9)	263(92.3)	71(84.5)	231(83.7)	143(88.3)	847(86.3)
Yes	35(20.1)	22(7.7)	13(15.5)	45(16.3)	19(11.7)	134(13.7)
Cognitive impairment; n (%)						
No	None	185(86.1)	67(87.0)	212(82.5)	141(83.9)	605(84.4)
Yes	None	30(13.9)	10(13)	45(17.5)	27(16.1)	112(15.6)
Malnutrition; n (%)						
No	157(89.2)	252(87.8)	64(77.1)	232(84.1)	150(86.2)	855(85.8)
Yes	19(10.8)	35(12.2)	19(22.9)	44(15.9)	24(13.8)	141(14.2)

IQR=interquartile range

### Antibody titres and features of epilepsy

There was significantly higher median antibody titer levels of HIV in severe epilepsy (optical density (OD)=0.14 (IQR: 0.10-0.15)) compared to non-severe epilepsy (0.13 (IQR: 0.11-0.18)) (P<0.001). There were lower median antibody titer levels of *Onchocerca volvulus* in severe epilepsy [optical density, OD=0.09 (IQR: 0.08-0.28)] compared to non-severe epilepsy [0.10 (IQR: 0.08-0.36)]; P=0.038. No significant differences for antibody tires to other infections were noted between severe and non-severe epilepsy ( Table 2). More than three quarters (88%) of the study population were exposed to some infection.

**Table 2.** Association between infection status and clinical and comorbidity features of epilepsy (pooled data).

Feature	Infection status		Crude Relative Risk (C.I)	P-value	Adjusted Relative Risk (C.I)	P-value
	Uninfected n (%)	Infected n (%)				
**Focal seizures** No Yes	64(11.7) 52(11.6)	482(88.3) 398(88.4)	1.08 (0.95–1.22)	0.247	0.97 (0.73–1.30)	0.873
**Abnormal EEG** No Yes	41(12.5) 49(12.5)	286(87.5) 342(87.5)	1.00 (0.90–1.11)	0.996	0.96 (0.83–1.11)	0.588
**Status epilepticus** No Yes	111(13.2) 5(3.3)	733(86.8) 148(96.7)	**2.79** **(2.53–3.08)**	**<0.001**	0.90 (0.32–2.54)	0.845
**Frequency of seizures** Monthly Daily/weekly	46(10.2) 15(7.3)	405(89.8) 190(92.7)	1.14 (0.56–2.31)	0.722	**2.85** **(1.95–4.16)**	**<0.001**
**Cognitive/learning difficulties** No Yes	31(5.1) 4(3.6)	574(94.9) 108(96.4)	1.39 (0.99–1.94)	0.059	**0.25** **(0.11–0.57)**	**0.001**
**Neurologic deficits** No Yes	102(12.0) 13(9.7)	745(88.0) 121(90.3)	**1.73** **(1.35–2.22)**	**<0.001**	1.40 (0.52–3.80)	0.509
**Malnutrition** Not malnourished Malnourished	95(11.1) 21(14.9)	760(88.9) 120(85.1)	0.71 (0.39–1.28)	0.258	0.91 (0.48–1.73)	0.772

C.I=confidence intervals; EEG=electroencephalograph

### Associations of parasitic and HIV infections with features of epilepsy among all participants

**Figure 1.**
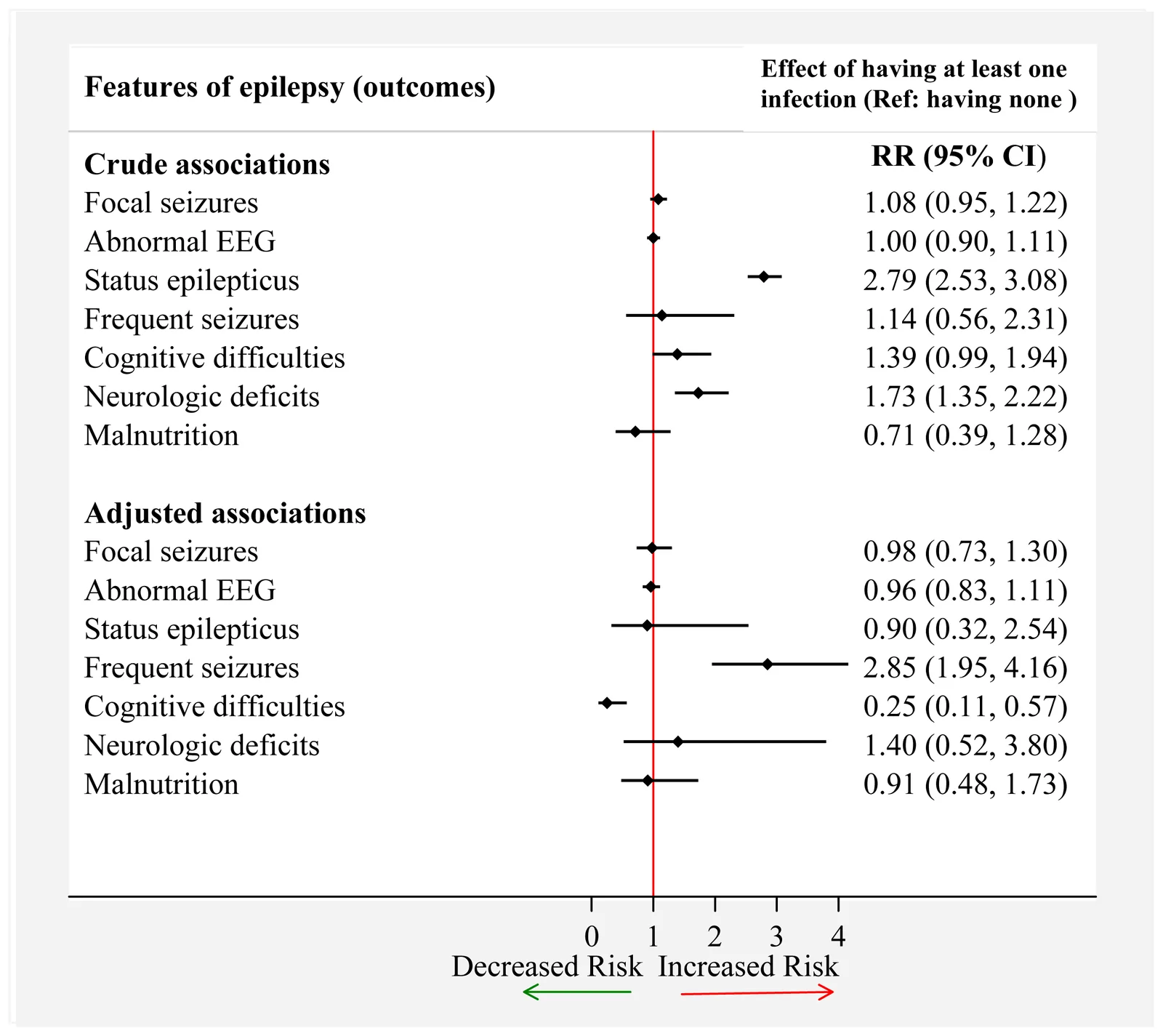
Associations between infections and epilepsy features in the study population (pooled). *Note: For avoidance of doubt, the features displayed in the plot are outcomes (dependent variables). The main predictor is parasitic/HIV infections (having any of these), adjusted for socio-demographics. Therefore, the effect size (relative risk (RR)) represents the effect of infections on a given feature, and not the other way round. EEG=electroencephalograph; CI=confidence interval*.

In the crude analysis, presence of infections as shown in Figure 1, was associated with convulsive status epilepticus (RR=2.79, 95% confidence interval (95%CI):2.53-3.08) and neurologic deficits (RR=1.73, 95%CI:1.35-2.22). After adjusting for age, sex, education and employment, presence of infections was associated with higher frequency (daily/weekly) of seizures (RR=2.85, 95%CI:1.95-4.16), and adjusting for these variables appeared to reduce risk for intellectual disability (RR=0.25, 95%CI:0.11-0.57) ( Figure 1). More detailed results also shown in Table 2.

In the sensitivity analysis of individual infections, exposure to falciparum malaria increased the risk of status epilepticus (RR=1.79, 95%CI:1.19-2.69), but reduced risk for several other features ( Table 3). HIV increased risk for seizure frequency (RR=1.16, 95%CI:1.07-1.27) and abnormal EEG (RR=1.30, 95%CI:1.01-1.69) among others. *O. volvulus* increased risk for convulsive status epilepticus (RR=2.32, 95%CI:1.35-3.98), *T. solium* for abnormal EEG (RR=1.54, 95%CI:1.31-1.82) and *T. canis* for neurological deficits (RR=1.69, 95%CI:1.53-1.85). Other associations are in Table 3.

**Table 3.** Adjusted associations of features of epilepsy and individual infections (pooled data).

Infections (predictors)	*P. falciparum* (+ve vs –ve) (ref: -ve)		HIV (+ve vs –ve) (ref: -ve)		*T. Gondii* (+ve vs –ve) (ref: -ve)		*O. Volvulus* (+ve vs –ve) (ref: -ve)		*T. solium* (+ve vs –ve) (ref: -ve)		*T. canis* (+ve vs –ve) (ref: -ve)	
Epilepsy features (outcomes)												
	**RR** **(C.I)**	**P–value**	**RR** **(C.I)**	**P–value**	**RR** **(C.I)**	**P–value**	**RR** **(C.I)**	**P–value**	**RR** **(C.I)**	**P–value**	**RR** **(C.I)**	**P–value**
**Focal seizure**	**0.33** **0.28–0.37**	**<0.001**	**1.14** **1.02–1.26**	**0.017**	1.05 0.81–1.37	0.691	**0.81** **0.71–0.93**	**0.003**	0.57 0.07–4.72	0.606	0.81 0.56–1.17	0.263
**Status epilepticus**	**1.79** **1.19–2.69**	**0.005**	**1.88** **1.21–2.93**	**0.005**	**0.71** **0.64–0.80**	**<0.001**	**2.32** **1.35–3.98**	**0.002**	0.83 0.24–2.80	0.759	1.28 0.29–5.56	0.743
**Frequent seizures**	0.85 0.55–1.31	0.459	**1.16** **1.07–1.27**	**0.001**	1.01 0.75–1.37	0.950	**0.76** **0.68–0.84**	**<0.001**	0.47 0.10–2.28	0.0.347	**1.27** **1.02–1.56**	**0.030**
**Cognitive difficulties**	**0.23** **0.21–0.26**	**<0.001**	**0.71** **0.58–0.88**	**0.001**	1.01 0.74–1.36	0.967	1.06 0.82–1.38	0.640	0.55 0.15–2.04	0.374	1.16 0.67–2.00	0.603
**Neurologic deficits**	**0.61** **0.46–0.82**	**0.001**	0.59 0.28–1.26	0.174	0.79 0.47–1.33	0.379	0.79 0.32–1.98	0.613	0.76 0.26–2.24	0.619	**1.69** **1.53–1.85**	**<0.001**
**Abnormal EEG**	**0.83** **0.69–0.99**	**0.041**	**1.30** **1.01–1.69**	**0.041**	1.19 0.94–1.51	0.150	0.93 0.69–1.26	0.639	**1.54** **1.31–1.82**	**<0.001**	0.96 0.84–1.09	0.517
**Malnutrition**	**0.79** **0.66–0.93**	**0.006**	1.33 0.39–4.56	0.652	0.84 0.62–1.14	0.258	0.94 0.73–1.21	0.654	**1.97** **1.18–3.27**	**0.009**	1.12 0.86–1.47	0.396

*Note: Frequent seizures – having seizures on a daily/weekly basis. EEG=electroencephalograph; RR=risk ratio; C.I=confidence intervals*

### Associations of parasitic/HIV infections with features of epilepsy among children

**Figure 2.**
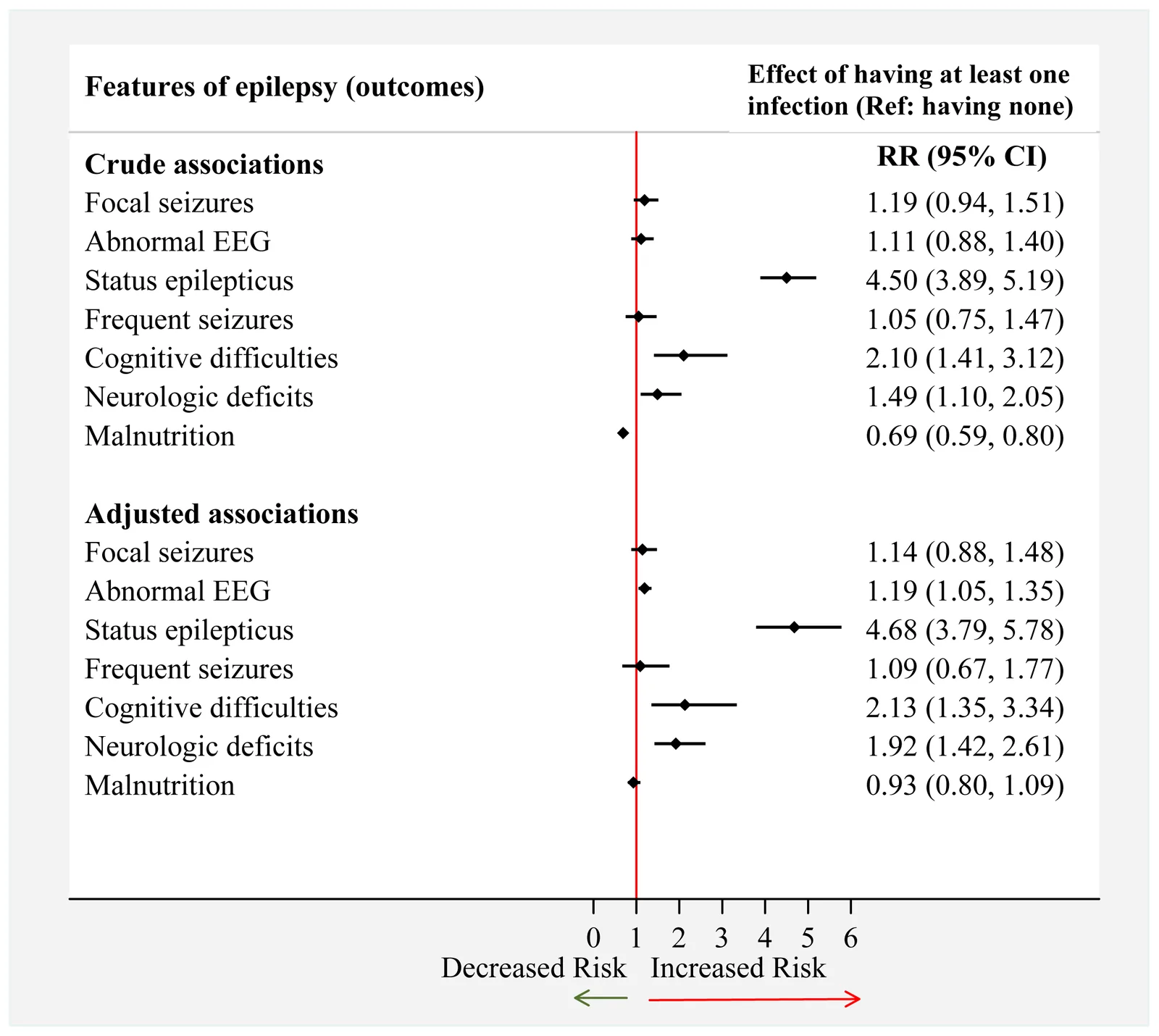
Associations between infections and epilepsy features in children (<18 years). *Note: For avoidance of doubt, the features displayed in the plot are outcomes (dependent variables). The main predictor is parasitic/HIV infections (having any of these), adjusted for socio-demographics. Therefore, the effect size (relative risk (RR)) represents the effect of infections on a given feature, and not the other way round. EEG=electroencephalograph; CI=confidence interval*.

In the crude analysis, exposure to parasitic/HIV infections was associated with convulsive status epilepticus (RR=4.50, 95%CI:3.89-5.19), intellectual disability (RR=2.10, 95%CI:1.41-3.12), neurologic deficits (RR=1.49, 95%CI:1.10-2.05) and with reduced risk for malnutrition (RR=0.69, 95%CI:0.59-0.80) ( Figure 2). When we adjusted for age and sex (majority had not reached the appropriate age for employment and school enrollment), presence of infections was associated with convulsive status epilepticus (RR=4.68, 95%CI:3.79-5.78), intellectual disability (RR=2.13, 95%CI:1.35-3.34) and neurologic deficits (RR=1.92, 95%CI:1.42-2.61). More detailed results are shown in Table 4.

**Table 4.** Association between infection status and clinical and comorbid features of epilepsy in children (< 18years).

Feature	Infection status		Crude Relative Risk (C.I)	P-value	Adjusted Relative Risk (C.I)	P-value
	Uninfected n (%)	Infected n (%)				
**Focal seizures** No Yes	34(14.2) 26(14.2)	205(85.8) 157(85.8)	1.19 (0.94-1.51)	0.155	1.14 (0.88-1.48)	0.324
**Abnormal EEG** No Yes	19(16.7) 23(13.5)	95(83.3) 148(86.5)	1.11 (0.88-1.40)	0.366	**1.19** **(1.05-1.35)**	**0.008**
**Status epilepticus** No Yes	56(16.8) 4(4.6)	278(83.2) 84(95.4)	**4.50** **(3.89-5.19)**	**<0.001**	**4.68** **(3.79-5.78)**	**<0.001**
**Frequency of seizures** Monthly Daily/weekly	15(9.3) 10(9.7)	146(90.7) 93(90.3)	1.05 (0.75-1.47)	0.783	1.09 (0.67-1.77)	0.740
**Cognitive/learning difficulties** No Yes	27(9.6) 2(4.4)	254(90.4) 43(95.6)	**2.10** **(1.41-3.12)**	**<0.001**	**2.13** **(1.35-3.34)**	**0.001**
**Neurologic deficits** No Yes	54(14.6) 5(10.2)	316(85.4) 44(89.8)	**1.49** **(1.10-2.05)**	**0.011**	**1.92** **(1.42-2.61)**	**<0.001**
**Malnutrition** Not malnourished Malnourished	47(13.0) 13(21.3)	314(87.0) 48(78.7)	**0.69** **(0.59-0.80)**	**<0.001**	0.93 (0.80-1.09)	0.392

EEG=electroencephalograph; C.I=confidence intervals

In the sensitivity analysis for individual infections, exposure to *P. falciparum* was significantly associated with having convulsive status epilepticus (RR=2.26, 95%CI:1.85-2.76), malnutrition (RR=1.59, 95%CI:1.20-2.11 *),* intellectual disabilities *(RR=0.15, 95%CI: 0.13-0.17)* and neurologic deficits (RR=0.47, 95%CI:0.33-0.68), after adjusting for age, sex and other infections ( Table 5). Exposure to *T. canis* was significantly associated with status epilepticus (RR=1.72, 95%CI:1.33-2.21) and neurologic deficits (RR=2.11, 95%CI:1.54-2.89) after adjusting for age, sex and other infections. Sero-positivity to *T. gondii* was protective for neurological deficits (RR=0.57, 95%CI:0.43-0.75) having controlled for age, sex and other infections ( Table 5).

**Table 5.** Adjusted associations of infections and features of epilepsy among minors (<18 years).

Infections (predictors)	*P. falciparum* (+ve vs –ve) (ref: -ve)		HIV (+ve vs –ve) (ref: -ve)		*T. Gondii* (+ve vs –ve) (ref: -ve)		*O. Volvulus* (+ve vs –ve) (ref: -ve)		*T. solium* (+ve vs –ve) (ref: -ve)		*T. canis* (+ve vs –ve) (ref: -ve)	
Epilepsy features (outcomes)												
	RR (C.I)	P-value	RR (C.I)	P-value	RR (C.I)	P-value	RR (C.I)	P-value	RR (C.I)	P-value	RR (C.I)	P-value
**Focal seizure**	0.90 0.68-1.21	0.510	0.64 0.19-2.13	0.467	**0.75** **0.68-0.82**	**<0.001**	0.90 0.37-2.24	0.837	0.82 0.14-4.69	0.822	0.99 0.49-2.00	0.986
**Status epilepticus**	**2.26** **1.85-2.76**	**<0.001**	0.44 0.03-5.96	0.539	0.51 0.14-1.80	0.327	**0.37** **0.18-0.75**	**0.006**	*No convergence*		**1.72** **1.33-2.21**	**<0.001**
**Frequent seizures**	0.80 0.42-1.53	0.506	**1.48** **1.07-2.06**	**0.019**	1.02 0.69-1.50	0.933	0.91 0.52-1.58	0.742	*No convergence*		**1.41** **1.03-1.94**	**0.033**
**Cognitive difficulties**	**0.15** **0.13-0.17**	**<0.001**	**0.32** **0.10-1.08**	**0.066**	0.89 0.72-1.10	0.275	1.54 0.55-4.29	0.409	**1.72** **1.48-2.01**	**<0.001**	1.28 0.68-2.40	0.444
**Neurologic deficits**	**0.47** **0.33-0.68**	**<0.001**	0.41 0.07-2.31	0.314	**0.57** **0.43-0.75**	**<0.001**	0.84 0.56-1.26	0.387	**2.81** **1.94-4.09**	**<0.001**	**2.11** **1.54-2.89**	**<0.001**
**Abnormal EEG**	**0.61** **0.46-0.81**	**0.001**	1.14 1.02-1.28	0.019	1.15 0.84-1.57	0.377	1.07 0.59-1.95	0.826	**1.79** **1.63-1.98**	**<0.001**	0.78 0.58-1.05	0.097
**Malnutrition**	**1.43** **1.01-2.04**	**0.046**	1.99 0.86-4.58	0.106	0.97 0.27-3.49	0.966	0.78 0.45-1.34	0.368	5.20 0.98-27.7	0.053	**1.35** **1.20-1.52**	**<0.001**

*Note: No convergence – the model would not produce parameter estimates due to inadequate cell frequencies; Frequent seizures – having seizures on a daily/weekly basis. EEG=electroencephalograph; RR=relative risk; C.I=confidence intervals*

### Associations of infections with features of epilepsy among adults

**Figure 3.**
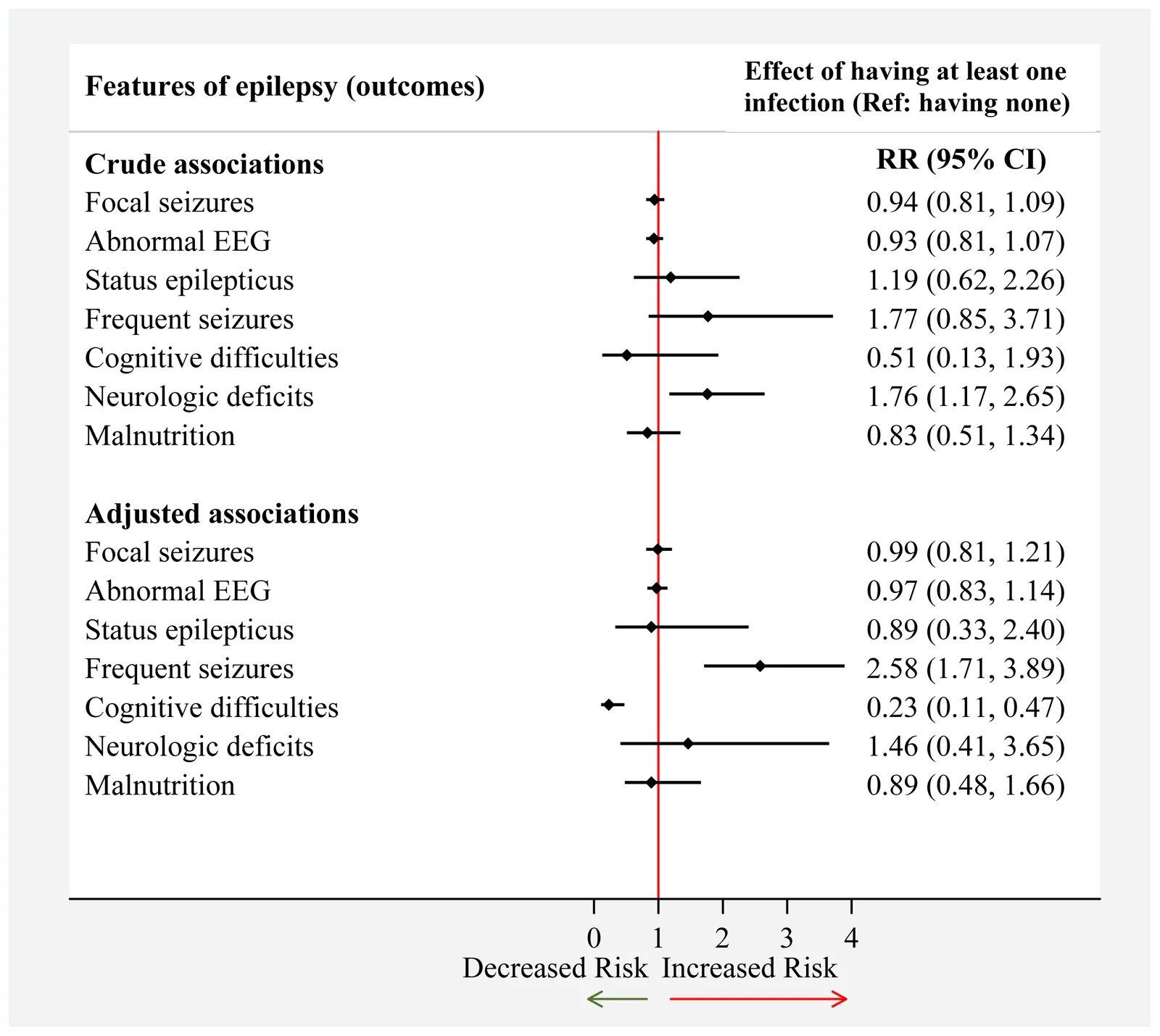
Association between infections and features of epilepsy in adults (≥18 years). *Notes: For avoidance of doubt, the features displayed in the plot are outcomes (dependent variables). The main predictor is Central Nervous System infections (having any of these), adjusted for socio-demographics. Therefore, the effect size (relative risk (RR)) represents the effect of infections on a given feature, and not the other way round. EEG=electroencephalograph; CI=confidence interval*.

Only neurological deficits (RR=1.76, 95%CI:1.17-2.65) was associated with presence of any infections in bivariate analysis. After adjusting for age, sex, education and employment, frequent seizures (RR=2.58, 95%CI:1.71-3.89) and cognitive difficulties (RR=0.23, 95%CI:0.11-0.47) were significantly associated with presence of infections ( Figure 3). More detailed results are shown in Table 6.

**Table 6.** Association between infection status and clinical and comorbid features of epilepsy in adults (≥18 years).

Feature	Infection status		Crude Relative Risk (C.I)	P-value	Adjusted Relative Risk (C.I)	P-value
	Uninfected n (%)	Infected n (%)				
**Focal seizures** No Yes	30(9.8) 26(9.7)	277(90.2) 241(90.3)	0.94 (0.81-1.09)	0.399	0.99 (0.81-1.21)	0.911
**Abnormal EEG** No Yes	22(10.3) 26(11.8)	191(89.7) 194(88.2)	0.93 (0.81-1.07)	0.301	0.97 (0.83-1.14)	0.739
**Status epilepticus** No Yes	55(10.8) 1(1.5)	455(89.2) 64(98.5)	1.19 (0.62-2.26)	0.600	0.89 (0.33-2.40)	0.814
**Frequency of seizures** Monthly Daily/weekly	31(10.7) 5 (4.9)	259(89.3) 97(95.1)	1.77 (0.85-3.71)	0.127	**2.58** **(1.71-3.89)**	**<0.001**
**Cognitive/learning difficulties** No Yes	4 (1.2) 2 (3.0)	320(98.8) 65(97.1)	0.51 (0.13-1.93)	0.322	**0.23** **(0.11-0.47)**	**<0.001**
**Neurologic deficits** No Yes	48 (10.1) 8 (9.4)	429(89.9) 77(90.6)	**1.76** **(1.17-2.65)**	**0.006**	1.46 (0.59-3.65)	0.414
**Malnutrition** Not malnourished Malnourished	48 (9.7) 8 (10)	446(90.3) 72(90)	0.83 (0.51-1.34)	0.437	0.89 (0.48-1.66)	0.716

EEG=electroencephalograph; C.I=confidence intervals

In the sensitivity analysis for individual infections, exposure to *P. falciparum* infection was significantly associated with convulsive status epilepticus (RR=1.74, 95%CI: 1.05-2.90) and frequent seizures (RR=3.07, 95%CI: 2.14-4.42), adjusting for age, sex, education and employment status. Additionally, after controlling for the confounders, infection with *O. volvulus* was significantly associated with status epilepticus (RR=2.31, 95%CI:1.36-3.93) and focal seizures (RR=0.80, 95%CI: 0.71-0.91). Further, infection with *T. solium* was significantly associated with malnutrition (RR=1.53, 95%CI:1.11-2.10) in the adjusted analysis ( Table 7).

**Table 7.** Adjusted associations of infections and features of epilepsy among adults (≥18 years).

Infections (predictors)	*P. falciparum* (+ve vs –ve) (ref: -ve)		HIV (+ve vs –ve) (ref: -ve)		*T. Gondii* (+ve vs –ve) (ref: -ve)		*O. Volvulus* (+ve vs –ve) (ref: -ve)		*T. solium* (+ve vs –ve) (ref: -ve)		*T. canis* (+ve vs –ve) (ref: -ve)	
Epilepsy features (outcomes)												
	**RR** **(C.I)**	**P–value**	**RR** **(C.I)**	**P–value**	**RR** **(C.I)**	**P–value**	**RR** **(C.I)**	**P–value**	**RR** **(C.I)**	**P–value**	**RR** **(C.I)**	**P–value**
**Focal seizures**	1.13 0.96–1.34	0.125	**1.12** **1.02–1.23**	**0.015**	1.05 0.81–1.35	0.719	**0.80** **0.71–0.91**	**0.001**	0.57 0.07–4.71	0.600	0.81 0.57–1.17	0.269
**Status epilepticus**	**1.74** **1.05–2.90**	**0.033**	**1.88** **1.22–2.91**	**0.05**	**0.71** **0.64–0.80**	**<0.001**	**2.31** **1.36–3.93**	**0.002**	0.83 0.25–2.81	0.768	1.28 0.28–5.89	0.749
**Frequent seizures**	**3.07 2.14–4.42**	**<0.001**	0.84 0.68–1.04	0.110	1.02 0.77–1.35	0.893	0.63 0.39–1.01	0.055	1.38 0.15–12.6	0.778	0.90 0.64–1.28	0.566
**Cognitive difficulties**	**0.42 0.26–0.70**	**0.001**	0.81 0.66–1.00	0.050	1.02 0.69–1.51	0.927	0.96 0.73–1.25	0.750	No convergence		1.19 0.77–1.83	0.443
**Neurologic deficits**	1.15 0.59–2.24	0.685	**0.68** **0.55–0.86**	**0.001**	0.84 0.40–1.76	0.644	0.77 0.29–2.04	0.603	No convergence		1.18 0.77–1.81	0.441
**Abnormal EEG**	0.97 0.78–1.21	0.772	1.46 0.93–2.30	0.102	1.17 0.80–1.70	0.418	**0.83** **0.73–0.95**	**0.0.005**	0.90 0.52–1.56	0.719	**1.25** **1.02–1.54**	**0.035**
**Malnutrition**	**0.68** **0.56–0.82**	**<0.001**	1.07 0.43–2.66	0.883	1.09 0.63–1.89	0.763	0.96 0.66–1.41	0.835	**1.53** **1.11–2.10**	**0.009**	1.19 0.86–1.64	0.300

*Note: No convergence – the model would not produce parameter estimates due to inadequate cell frequencies; Frequent seizures – having seizures on a daily/weekly basis. EEG=electroencephalograph; RR=relative risk; C.I=confidence intervals*

### Interaction between infections and features of epilepsy

In the analysis for multiplicative interactions, several paired infections showed interactions ( Table 8 and supplementary table 1). In particular, *P. falciparum* and HIV interacted to increase the risk for convulsive status epilepticus (RR=2.35, 95%CI:1.38-4.00) and frequent seizures (RR=4.14, 95%CI:3.04-5.65), but the interaction reduced the risk for cognitive impairment (RR=0.32, 95%CI: 0.24-0.44). Similarly, HIV interacted with *O. volvulus* to increase the risk for convulsive status epilepticus (RR=4.46, 95%CI:2.05-9.71), but reduced the risk for frequent seizures (RR=0.74, 95%CI:0.63-0.88).

**Table 8.** Statistical (multiplicative) interactions of infections and epilepsy features.

	*P. falciparum*	HIV	*T. gondii*	*O. volvulus*	*T. solium*	*T. canis*
***P. falciparum***						
**HIV**	Status epilepticus ^**+**^ Frequent seizures ^**+**^ Cognitive difficulties ^**-**^ Neurological deficits ^**-**^					
***T. gondii***	Focal seizures ^**-**^ Status epilepticus ^**+**^ Frequent seizures ^**+**^ Cognitive difficulties ^**-**^	Abnormal EEG ^**+**^				
***O. volvulus***	Focal seizures ^**-**^ Cognitive difficulties ^**-**^ Abnormal EEG ^**-**^	Focal seizures ^**-**^ Status epilepticus ^**+**^ Frequent seizures ^**-**^ Neurologic deficits ^**-**^	NONE			
***T. solium***	NONE	NONE	NONE	Abnormal EEG ^**+**^		
***T. canis***	Focal seizure ^**-**^ Frequent seizures ^**+**^ Cognitive difficulties ^**-**^ Neurological deficits ^**+**^ No ASM use ^**+**^	Abnormal EEG ^**+**^	NONE	Focal seizures ^**-**^ Frequent seizures ^**-**^	NONE	

*Notes: ^+^Interaction increases risk; ^-^Interaction reduces risk*; ASM=anti-seizure medication

In the analysis for biological interactions, *T. gondii* and *T. canis* interacted synergistically to increase the risk of convulsive status epilepticus (RERI = 0.92, 95%CI:0.46-1.38) while *P. falciparum* interacted with HIV to reduce the risk of convulsive status epilepticus (RERI = -4.02, 95%CI:-7.95 - -0.08) ( Table 9). Detailed results for biological interactions are shown in supplementary table 2, while those for population attributable fractions are in supplementary table 3.

**Table 9.** Additive (biological) interactions of parasitic/HIV infections in the risk for epilepsy features.

	*P. falciparum*	HIV	*T. gondii*	*O. volvulus*	*T. solium*	*T. canis*
***P. falcip***						
**HIV**	Status epilepticus ^-^					
***T. gondii***	NONE	NONE				
***O. volvulus***	NONE	Abnormal EEG ^-^	Status epilepticus ^+^			
***T. solium***	NONE	NONE	Cognitive difficulties ^-^	NONE		
***T. canis***	No ASM use ^-^	NONE	NONE	NONE	NONE	

*Notes: ^+ ^synergism; ^- ^antagonism*; ASM=anti-seizure medication

## Discussion

This study provides important evidence that parasitic infections that infect the CNS are not only associated with epilepsy, but also determine the occurrence of features and phenotypes of epilepsy. In children with epilepsy, exposure to infections is particularly associated with convulsive status epilepticus, neurological deficits and intellectual disability, while in adults it is associated with frequent seizures. Individual infections may increase some features (e.g. falciparum malaria increasing risk for convulsive status epilepticus) but not others. These infections may interact synergistically or antagonistically to affect the features of epilepsy, and their prevention may reduce up to 26% severe epilepsy.

### Antibody titres and prevalence for parasitic/HIV infections

There was significant difference in median antibody titer levels between severe and non-severe epilepsy only for HIV (P<0.001) and *Onchocerca volvulus* (P=0.038), suggesting that these levels are ubiquitous representation of exposure, and reliable associations should be measured from cut-offs derived from high antibody titres. Using predefined cut-offs, infections were more prevalent across all features considered, indicating that these infections could be potential risk factors for development of these epilepsy features. The prevalence of infections were significantly greater than in the general population since they are based on an epilepsy cohort, whose exposure to infections is already known to be relatively greater ( Carter *et al*., 2004).

### Associations between any parasitic/HIV infections and features of epilepsy in all populations

Among all the study population, exposure to any of the infections were associated with increased risk of having frequent (daily/weekly) seizures and an increased risk of cognitive difficulties, after controlling for confounding effects, including the use of ASM. HIV-positive individuals have increased risk of abnormal EEG, which has been noted in previous studies ( Kellinghaus *et al*., 2008). *T. canis* was associated with neurological deficits which is not surprising since neurological involvement has been reported ( Finsterer & Auer, 2007). Associations that appeared protective were observed especially for some specific infections. This may be due to absence of these features and phenotypes when samples for positive assays were collected or may be a spurious association. Since susceptibility to infections and pathogenesis of epilepsy following these infections is different between children and adults, it warranted evaluations of these associations between two population strata i.e. adults and children/minors separately.

### Associations between any parasitic/HIV infections and features of epilepsy in children

Among children with epilepsy, we found that exposure to infections was associated with higher risk of having status epilepticus, intellectual disabilities and neurologic deficits after adjusting for age and sex. Analysis of individual infections showed that *P. falciparum* increased the risk for convulsive status epilepticus, and *T. canis* that for neurological deficits, while interaction of several infections may explain the intellectual disability.

Children have low immunity to infections and also have a developing brain, and it is not surprising that infections determine three features of epilepsy that are pathognomonic of severe childhood seizure disorders. Convulsive status epilepticus is common in children and previous studies showed that malaria is the most important cause ( Sadarangani *et al*., 2008). Kariuki *et al*. in 2011 hypothesized that the non-malarial status epilepticus that did not decline over a period of malaria reduction was probably caused by epilepsy ( Kariuki *et al*., 2011). It has been established in a recent multisite study that status epilepticus occurs in over 25% of people with epilepsy ( Kariuki *et al*., 2015a), and infections may contribute to this excess burden of prolonged seizures.

### Associations between any parasitic/HIV infections and features of epilepsy in adults

Among adults with epilepsy, exposure to any infection was associated with increased risk for frequent seizures, but reduced risk for intellectual disability. Analysis of individual impairments shows that the increased risk for frequent seizures, status epilepticus was determined by falciparum malaria, which appeared to reduce the risk for intellectual disability in adults, perhaps suggesting these are related to other causes. Status epilepticus was also increased following *O. volvulus*, with other infections showing protection against some of these features.

The positive association of infections with frequent seizures is expected, since we have demonstrated in children that repetitive seizures are highly attributable to malaria ( Kariuki *et al*., 2011). Presence of any infections were protective for intellectual disability in adults with epilepsy perhaps because of absence or low frequency of infections in this group of people movement of adults with cognitive difficulties is restricted indoors, resulting in less exposure to infections. Alternatively, it may be due to reporting bias whereby adults with epilepsy conceal their cognitive impairments, which is easily recognized as a condition for children ( Carter *et al*., 2005) or due to counselling out of effect sizes of infections that confers susceptibility by those that reduces risk.

### Association of individual parasitic/HIV infections with features of epilepsy

When we probed the effects of individual infections on the features of epilepsy, *P. falciparum* was protective for most epilepsy features, except for status epilepticus. The same case applied to children, but in adults, *P. falciparum* infection was associated with increased risk of both status epilepticus and daily/weekly seizures. This finding agrees with that of another study done in Africa, where infection with falciparum malaria were important risk factors for status epilepticus ( Kariuki *et al*., 2015a). We also found that exposure to falciparum malaria may in part determine the risk for malnutrition in those with epilepsy, supporting the previous association between malaria and malnutrition in general hospital admissions ( Gone *et al*., 2017), but other factors involved in this association should be considered. HIV was a risk factor for frequent daily/weekly seizures, and abnormal EEG, suggesting it has neurological involvement. Seizures have been reported in HIV with or without epilepsy ( Kellinghaus *et al*., 2008); frequent seizures in our study are likely due to epileptogenic nature of HIV since its negative association with neurological deficits appears to rule out contribution by brain damage. *O. volvulus* was protective for focal seizures and frequent seizures but increased the risk for status epilepticus. *O. volvulus* likely has diffuse brain involvement resulting in convulsive status epilepticus with a generalized onset in the brain, thus the negative association with focal seizures. *Taenia solium* increased the risk of having abnormal EEG but reduced the risk of early onset seizures. The finding that *T. solium* appeared to reduce the risk of early onset seizures may be because it is more common in adult onset epilepsy ( Ngugi *et al*., 2013).

### Interaction of parasitic/HIV infections and features of epilepsy

More interactions were noted in the additive scale than in the multiplicative scale, but the later would only be helpful in examining the contribution of a risk factor to a disease. Interaction on the additive scale has public health importance in that it is easier to quantify the frequency of a disease that would be controlled by the removal of risk factors. The many significant combinations of interaction in the multiplicative scale suggests that multiple infections determine the features and phenotypes of epilepsy in Africa. Interaction tests performed on additive scale revealed that infections interact either synergistically or antagonistically in influencing the features of epilepsy. For instance, *Toxoplasma gondii* and *Toxocara canis* interacted synergistically to increase the risk of status epilepticus, implying that the number of status epilepticus cases due to the coinfection is higher than the total number of cases contributed by the two infections separately. In contrast, *P. falciparum* and HIV interacted antagonistically towards status epilepticus, implying that co-infections worsen the prognosis of epilepsy, and can also be found in those with milder features. These interaction findings support the need to screen for multiple infections in people with epilepsy.

### Strengths and limitations

Strengths: This study had more than the minimum required sample size, meaning the study had enough power to answer our research questions. Also, being a multisite design, the findings can be generalized across ecological zones considered. Additionally, the mixed effects model used in this study took accounted for the correlations (homogeneity) within sites and heterogeneity across sites. Limitations: We only studied active convulsive epilepsy. The infections were assayed for exposure and not really a measure of impact of severe disease on epilepsy, which would be done with longitudinal cohort studies. Another cause of concern was the possibility of recall bias since participants were interviewed for some epilepsy features and the inherent challenge of inferring direction of causality for cross-sectional studies. Unmeasured risk factors could contribute to the association between infections and epilepsy features i.e. residual confounding. 

## Conclusions

Features and phenotypes of epilepsy in Africa may be determined by infections and the direction of association depends on the type, frequency of infection and their interaction. Exposure to parasitic infections determine different types of features and phenotypes for children and adults, with status epilepticus and cognition being important in children and frequent seizures in adults. Taken together these findings suggests that not only are infections associated with epilepsy, they also determine the presentation of epilepsy in terms of features and phenotypes. Control of infections may reduce the risk of epilepsy becoming severe in addition to preventing epilepsy directly caused by infections in the community.

## Previous publications

Part of this data was published in *PLoS Negl Trop Dis* ( https://www.ncbi.nlm.nih.gov/pmc/articles/PMC4038481/) and in *Lancet Neurol* ( https://www.ncbi.nlm.nih.gov/pmc/articles/PMC3581814/) as case-control studies to examine exposure to infections as risk factors for developing epilepsy. However, none of these two articles explored the role of exposure to infections in the occurrence of features, outcomes and phenotypes among people with epilepsy, which is now presented in this paper. More details on justification for the current analysis is provided in the introduction section of the paper.

## Data availability

### Underlying data

Harvard Dataverse: Infections and Epilepsy. https://doi.org/10.7910/DVN/WQZBUX ( Langat *et al*., 2021).

This project contains the following underling data:

(i) epilepsydata_deidentified_ID.tab (used for calculating the association of exposure to infections with epilepsy features and phenotypes)

For ethical considerations and to comply with the language in the informed consent forms, the access to the dataset is controlled for general research use. Please request to access the data by completing the forms in the provided link to the data, following which permissions will be granted using procedures governed by the Data Governance Committee (DGC) of the Centre for Geographic Medicine Research Coast, Kenya Medical Research Institute. Data will be made available with the approval of the KEMRI Wellcome Trust Research Programme Data Governance Committee, only where anonymization can be adequately achieved to protect the privacy and confidentiality of the participants/respondents and any mentioned individuals and institutions, and where the proposed use is seen as relevant to the nature of the data. Where the DGC recommend this, the national KEMRI Science and Ethics Review Unit may also be asked to approve the proposed use. Conditions for data sharing are outlined in a KWTRP Data Sharing Agreement, including that the requestor shall use the data only for the agreed purpose as stipulated in the application form; shall agree to keep the data strictly confidential and shall not in any way attempt to seek to discover the identity of data subjects, to compromise or infringe on their privacy and confidentiality of their information.

### Extended data

Harvard Dataverse: Infections and Epilepsy. https://doi.org/10.7910/DVN/WQZBUX ( Langat *et al*., 2021).

This project contains the following extended data:

(i) Infections and Epilepsy_Codebook.xlsx (description of variables codes and labels)
(ii) Infections Epilepsy Data Readme File.txt (brief description of the study aim and processes)
(iii) Infections and Epilepsy.do (SATA v15 analysis script)
(iv) StageIII SEEDS-Clinical Examination.doc (clinical examination form)
(v) StageIII SEEDS-Clinical History (Twi).doc (clinical history form)
(vi) StageIII SEEDS-EEEG.doc (EEG interpretation form)
(vii) StageIII SEEDS-Socio-Demography Above 18(Twi).doc (adults’ sociodemographic and medical history form)
(viii) StageIII SEEDS-Socio-Demography Under 18(Twi).doc (children’s sociodemographic and medical history form)
(ix) Supplementary materials_19July2021.docx (supplementary appendix, figure and tables).

Data are available under the terms of the Creative Commons Attribution 4.0 International license (CC-BY 4.0).
