# Supplementary material for: Exposure to parasitic infections is associated with features and phenotypes of active convulsive epilepsy in Africa
Source: Wellcome Open Res. 2026 Aug 10;6:200. Originally published 2021 Aug 5. [Version 3] doi: 10.12688/wellcomeopenres.17049.3 (PMC13560818; doi:10.12688/wellcomeopenres.17049.3)
Supplement: Peer review history

# Reviewer report: Reviewer response for version 3

**DOI:** 10.21956/wellcomeopenres.30210.r163977

**Published:** 2026-08-11

**Authors:** Yuelian Sun

**Affiliations:** 1 Department of Neurology, Aarhus University Hospital, Aarhus, Denmark

Thank you for the update. I have no further comments.

Is the work clearly and accurately presented and does it cite the current literature?

Yes

If applicable, is the statistical analysis and its interpretation appropriate?

No

Are all the source data underlying the results available to ensure full reproducibility?

No

Is the study design appropriate and is the work technically sound?

No

Are the conclusions drawn adequately supported by the results?

No

Are sufficient details of methods and analysis provided to allow replication by others?

No

Reviewer Expertise:

Epidemiologic research on risk factors, especially risk factors in early life for neurologic disorders, including epilepsy.

I confirm that I have read this submission and believe that I have an appropriate level of expertise to confirm that it is of an acceptable scientific standard.

# Reviewer report: Reviewer response for version 2

**DOI:** 10.21956/wellcomeopenres.19939.r51192

**Published:** 2022-07-25

**Authors:** Yuelian Sun

**Affiliations:** 1 Department of Neurology, Aarhus University Hospital, Aarhus, Denmark

I agreed with the author's arguments. However, the authors still use some strong words indicating 'causal inference' like 'Features and phenotypes of epilepsy in Africa may be determined by infections' in the conclusion, which could not be concluded from the findings of this study. Try to modify the words to reflect what the findings could show.

Is the work clearly and accurately presented and does it cite the current literature?

Yes

If applicable, is the statistical analysis and its interpretation appropriate?

No

Are all the source data underlying the results available to ensure full reproducibility?

No

Is the study design appropriate and is the work technically sound?

No

Are the conclusions drawn adequately supported by the results?

No

Are sufficient details of methods and analysis provided to allow replication by others?

No

Reviewer Expertise:

NA

I confirm that I have read this submission and believe that I have an appropriate level of expertise to confirm that it is of an acceptable scientific standard, however I have significant reservations, as outlined above.

# Response

**Published:** 2026-08-01

**Authors:** Symon Kariuki

**Affiliations:** Research Division, African Population and Health Research Center, Nairobi, Nairobi County, Kenya

**Response to reviewer (Dr Yuelian Sun) report** I agreed with the author's arguments. However, the authors still use some strong words indicating 'causal inference' like 'Features and phenotypes of epilepsy in Africa may be determined by infections' in the conclusion, which could not be concluded from the findings of this study. Try to modify the words to reflect what the findings could show.

**Response: Thank you for reading the revised article and supporting our conclusions. We agree that using the term “determine” may imply causal inference, which cannot be established in a cross-sectional analysis like ours. We have therefore revised the discussion section to use the phrase “associated with” instead of “determined”.**

# Reviewer report: Reviewer response for version 1

**DOI:** 10.21956/wellcomeopenres.18830.r50872

**Published:** 2022-06-13

**Authors:** Hui Jan Tan

**Affiliations:** 1 Department of Internal Medicine, Universiti Kebangsaan Malaysia Medical Centre (UKMMC), Kuala Lumpur, Malaysia

The article is well written and addresses the objectives succinctly. The authors provided good analytical data for the work. The data presented provided valuable insight to the gravity of the problems encountered. 

**Few comments:**

1. Was the multilogistic regression analysis done for the predictors?
2. Was there any active clinical infection for the seropositivity serology?
3. For the neurophysiology features: apart from focal seizures, was generalised seizures analysed?

Is the work clearly and accurately presented and does it cite the current literature?

Yes

If applicable, is the statistical analysis and its interpretation appropriate?

Yes

Are all the source data underlying the results available to ensure full reproducibility?

Yes

Is the study design appropriate and is the work technically sound?

Yes

Are the conclusions drawn adequately supported by the results?

Yes

Are sufficient details of methods and analysis provided to allow replication by others?

Yes

Reviewer Expertise:

epilepsy

I confirm that I have read this submission and believe that I have an appropriate level of expertise to confirm that it is of an acceptable scientific standard.

# Response

**Published:** 2022-06-16

**Authors:** Symon Kariuki

**Affiliations:** Research Division, African Population and Health Research Center, Nairobi, Nairobi County, Kenya

The article is well written and addresses the objectives succinctly. The authors provided good analytical data for the work. The data presented provided valuable insight to the gravity of the problems encountered.

**Reply: Thank you very much for positively appraising our manuscript. The useful concerns you raised are addressed below.**

1. Was the multilogistic regression analysis done for the predictors?

**Reply:** **Yes, a multivariable regression models were built, with all predictors, and exposure to infections as the response or outcome variable. Since exposure to infections was common in our sample, the odds ratios from a logistic regression model would overestimate the relative risks. This prompted use of multivariable modified Poisson regression models that produces accurate relative risks when outcomes are common in the population.**


** **

1. Was there any active clinical infection for the seropositivity serology?

**Reply:** **The samples for these assays were collected when all participants reported to have been clinically well. However, it is possible that persistent exposure to the infections e.g. falciparum malaria could lead to active infections given that previous hospitalizations with clinical malaria was reported in 5.3% persons with epilepsy from the primary epidemiological study that generated these samples** ^**1**^ **.**


** **

1. For the neurophysiology features: apart from focal seizures, was generalised seizures analysed?

**Response:** **Both focal and generalized electroencephalographic seizures were documented. The analyses considers both focal and generalized electroencephalographic seizures, whereby the variables “focal seizures” has those with focal features coded as “1” and those with generalized features as “0”** **^2^.**

**References**

 1.         Ngugi AK, Bottomley C, Kleinschmidt I, et al. Prevalence of active convulsive epilepsy in sub-Saharan Africa and associated risk factors: cross-sectional and case-control studies. *The Lancet Neurology* 2013; **12**(3): 253-63.

 2.         Kariuki SM, Matuja W, Akpalu A, et al. Clinical features, proximate causes, and consequences of active convulsive epilepsy in Africa. *Epilepsia* 2014; **55**(1): 76-85.

# Reviewer report: Reviewer response for version 1

**DOI:** 10.21956/wellcomeopenres.18830.r50157

**Published:** 2022-05-13

**Authors:** Yuelian Sun

**Affiliations:** 1 Department of Neurology, Aarhus University Hospital, Aarhus, Denmark

The authors aimed to examine if exposure to common central nervous system (CNS) infections may play a role in the early onset of epilepsy, convulsive status epilepticus, frequent seizures, abnormal electroencephalograph (EEG) findings, intellectual disabilities, neurological deficits as well as other medical comorbidities of epilepsy e.g. malnutrition.

 The population consisted of 1,711 patients diagnosed with epilepsy in the five African countries. 997 patients were defined as respondents for test of infection. Respondents were tested for *Toxacara canis*, *Onchocerca volvulus*, *Plasmodium falciparum*, *Toxoplasma gondii*, HIV and *Taenia solium* (exposed). infection status was determined through analysis of blood samples to detect antibodies to the infectious parasites and HIV. These assays to the infectious parasites and HIV were performed on 986 people with epilepsy.

 The main concern of this study is that the authors might be unable to make causal inference (between parasitic infections and risk of epilepsy) using the data they had.

 Although these parasitic infections could affect the central nervous system, they could not be defined as central nervous system (CNS) infections.

 Even for a descriptive study, for example, parasitic infections among these epilepsy cases, the authors should be aware of the representativeness of the 1,711, response rate (why some cases responded and some did not), and differences among countries.

**Minor comments:**

- In the background of the abstract, should ‘epilepsy foutcomes’ be ‘epilepsy outcomes’?
- Table 2. The total number, for example according to feature of focal seizure, is 996. How about those with missing value?

Is the work clearly and accurately presented and does it cite the current literature?

Yes

If applicable, is the statistical analysis and its interpretation appropriate?

No

Are all the source data underlying the results available to ensure full reproducibility?

No

Is the study design appropriate and is the work technically sound?

No

Are the conclusions drawn adequately supported by the results?

No

Are sufficient details of methods and analysis provided to allow replication by others?

No

Reviewer Expertise:

Epidemiologic research on risk factors, especially risk factors in early life for neurologic disorders, including epilepsy.

I confirm that I have read this submission and believe that I have an appropriate level of expertise to state that I do not consider it to be of an acceptable scientific standard, for reasons outlined above.

# Response

**Published:** 2022-06-16

**Authors:** Symon Kariuki

**Affiliations:** Research Division, African Population and Health Research Center, Nairobi, Nairobi County, Kenya

The authors aimed to examine if exposure to common central nervous system (CNS) infections may play a role in the early onset of epilepsy, convulsive status epilepticus, frequent seizures, abnormal electroencephalograph (EEG) findings, intellectual disabilities, neurological deficits as well as other medical comorbidities of epilepsy e.g. malnutrition.

 The population consisted of 1,711 patients diagnosed with epilepsy in the five African countries. 997 patients were defined as respondents for test of infection. Respondents were tested for *Toxacara canis*, *Onchocerca volvulus*, *Plasmodium falciparum*, *Toxoplasma gondii*, HIV and *Taenia solium* (exposed). infection status was determined through analysis of blood samples to detect antibodies to the infectious parasites and HIV. These assays to the infectious parasites and HIV were performed on 986 people with epilepsy.

 The main concern of this study is that the authors might be unable to make causal inference (between parasitic infections and risk of epilepsy) using the data they had.

**Response** **: Thank you for this important observation and agree that cross-sectional studies may not be useful in making causal inference, since the prevalence of seropositivity may vary if measured at a slightly different than when we did, and because it is difficult to pinpoint when the exposure occurred. However, cross-sectional studies, which easy and inexpensive, may be very useful generation of new or important hypothesis for understanding pathophysiology or prognosis or health conditions when this is not previously studied or understood** **^1^** **. For instance, many previous studies only explored the etiological role of infections in development of new onset epilepsy, with no literature on how the persistent exposure after development of epilepsy changes the course of the disease, including even increasing the odds for severe disability and premature mortality. In addition, the analytical approach in this cross-sectional study aims at computing prevalence ratios that approximates relative risks in cohort studies, when outcomes are common and short-lived e.g. acute infections** **^2^** **. Findings from this cross-sectional study are likely to inform set up of cohort studies to explore differential progression of epilepsy among persons exposed to infections, and to inform mass prophylaxis against exposure to infections among people already living with epilepsy. These observations are included in the statistical analysis section, limitations section and conclusion of the revised manuscript.**

 Although these parasitic infections could affect the central nervous system, they could not be defined as central nervous system (CNS) infections.

**Response:** **We agree that some parasites e.g. *Onchocerca volvulus* might not directly affect the brain, but rather it is the inflammation** **provoked by the death of the parasite and the subsequent release of Wolbachia, for example** **^3^** **, that may impact the brain indirectly. We have therefore revised the term “central nervous system (CNS) infections” to “infections with a propensity for the** **central nervous system (CNS)** **”, which we hope is more acceptable.**

 Even for a descriptive study, for example, parasitic infections among these epilepsy cases, the authors should be aware of the representativeness of the 1,711, response rate (why some cases responded and some did not), and differences among countries.

**Response:** **We agree that selection bias may be introduced by non-participation of participants. Some of those participated in the follow up evaluations may have had severe forms of epilepsy than those who dropped out, introducing severity bias which affect the associations that premised on severe forms of epilepsy as outcomes. Although there are many reasons for choosing not to participate in follow ups evaluations e.g. non-preference for biomedical facilities, other possibilities may be that some participants were unwell or even died, which can introduce survival bias. These possibilities are acknowledged in the limitation section of the revised manuscript.**

**Minor comments:**

- In the background of the abstract, should ‘epilepsy foutcomes’ be ‘epilepsy outcomes’?

**Response** **: Sorry for this typographical error, which has been revised as suggested.**


** **

- Table 2. The total number, for example according to feature of focal seizure, is 996. How about those with missing value?

**Response: The variable “focal seizures” includes only those with these data, and therefore those with missing information are not included in the analysis.**

**References**

 1.         Levin KA. Study design III: Cross-sectional studies. *Evidence-Based Dentistry* 2006; **7**(1): 24-5.

 2.         Zou G. A modified poisson regression approach to prospective studies with binary data. *American journal of epidemiology* 2004; **159**(7): 702-6.

 3.         Galán-Puchades MT. Onchocerciasis-associated epilepsy. *The Lancet Infectious Diseases* 2019; **19**(1): 21-2.
